# Evaluation of Strength Properties of Sand Stabilized with Wood Fly Ash (WFA) and Cement

**DOI:** 10.3390/ma15093090

**Published:** 2022-04-24

**Authors:** Sanja Dimter, Martina Zagvozda, Tea Tonc, Miroslav Šimun

**Affiliations:** 1 Josip Juraj Strossmayer University of Osijek, Faculty of Civil Engineering and Architecture Osijek, 31000 Osijek, Croatia; mzagvozda@gfos.hr (M.Z.); ttonc15@gfos.hr (T.T.); 2 Zagreb University of Applied Sciences, Civil Engineering Department Zagreb, 10000 Zagreb, Croatia; msimun@tvz.hr

**Keywords:** wood fly ash (WFA), sand, cement stabilized mixtures, mechanical properties

## Abstract

The article describes the laboratory evaluation of mixtures of sand modified with wood fly ash (WFA) and additionally stabilized with different amounts of cement. Laboratory research includes determining the California Bearing Ratio (CBR), compressive and indirect tensile strengths of the mixtures, and the resistance of mixtures to freezing/thawing cycles. The aim of the research is to determine if WFA, an alternative material, can improve sand bearing capacity and contribute to strength development while reducing necessary cement amounts and satisfying the technical regulation for use in pavement base courses. The test results obtained show that WFA has a considerable stabilization effect on the sand mixture and improves its load bearing capacity. By adding a small quantity of the cement, the hydraulic reaction in the stabilized mixture is more intense and results in greater strengths and an improved resistance to freezing. The test results show that, by replacement of part of the sand with WFA (in the quantity of 30%), greater strengths can be achieved in relation to the mixture of only sand and cement. Additionally, the content of cement necessary for the stabilization of sand (usually 8–12%) is considerably reduced, which enables cost savings in the construction of pavement structures.

## 1. Introduction

Load-bearing layers stabilized by cement or other hydraulic binders (fly ash, slag) are widely applied in pavement structures of roads throughout Europe and the world [1]. These layers take the static and dynamic traffic loads from the pavement surface and transfer them to the substructure. Cement stabilized layers with their mechanical properties must meet the stresses of traffic load and durability requirements during the design period of construction. They are carried out in asphalt pavement structures, in which they act as one of the most important elements in terms of load-bearing capacity, as well as in concrete pavement structures in which, as a base under the concrete slab, they prevent the occurrence of material “pumping”. The pavement structures that have cement stabilized layers in their composition are less sensitive to seasonal influences, their load bearing capacity is considerably more uniform, and they withstand heavy traffic loads.

Although there are several different definitions and names of mixtures for the construction of cement stabilized layers [1,2], the simplified one defines the cement stabilized mixture as a mixture of different types of granular material to which cement is added for the purpose of improving specific properties. For the production of cement-stabilized bearing layers, granular stone material (natural sandy gravel, crushed stone material, or their mixtures) or sand is used as the basic aggregate. The use of sandy gravel and the crushed stone aggregate also results in better mechanical properties of stabilization mixtures, while sandy materials produce mixtures with somewhat lower mechanical properties. Depending on the type of the aggregate and the desired mechanical properties of the stabilization mixture, cement is added to the stabilization mixture in the content of 3–12% [1,2,3]. By adding cement, the stabilized mixture increases in compressive and tensile strength, moduli of elasticity, the resistance to change of humidity, and the resistance to freezing and thawing cycles [1,3]. Thus, the improved properties and the increased load bearing capacity of the mixture make the cement stabilized mixture indispensable in the construction of pavement structures for heavy and very heavy traffic loads.

In addition to the mechanical properties of the material during the construction of pavement structures, the design and the selection of material for its execution and the cost-effectiveness, sustainability, and environmental impact [4] should also be taken into account. In designing cost-effective pavement structures, the accessibility and availability of local materials, of natural or industrial origin, are of great importance.

Thus, in the area of eastern Croatia, unlike other parts of Croatia, large amounts of river and dug sand are used in road construction. Sands are used for the construction of embankments, bedrock, and in the construction of unbound and cement-bound load-bearing layers of the pavement structure. The application of river and dug sands in eastern Croatia was imposed as a necessity in order to rationalize the costs of construction and was confirmed in the *Study of the possibilities of application of sand in the construction of roads of Slavonia and the Baranya region* [5]. Sand is applied in the natural state or stabilized with cement. The content of cement necessary for stabilization of sand is increased in relation to other granular stone materials and it amounts to 8–12%. Sand application as a local material enabled savings of as much as 50–60% in costs of transport of granular stone material. Through many years of application in road construction, sand has proven to be a quality and suitable local engineering material in the design and construction of cost-effective pavement structures and embankment layers [6].

Besides natural local materials, waste materials and industrial by-products also play a considerable role in the construction of cost-effective pavement structures [7,8]. These are materials that require complex depositing of large quantities at dump sites and are, as such, harmful for the environment, whilst their application has a significant economic justification and is clearly encouraged by sustainable development guidelines [4]. Ash from wood biomass (WA) is one of the industrial by-products with great potential for application in civil engineering, especially in road construction [4,8,9], where finer and coarser fractions of ash can be implemented in all layers of the pavement structure. WA is characterized as non-hazardous waste and is formed as a residue from the combustion of wood biomass for the production of electricity and heat. Based on the place of their creation and accumulation, WA are divided into three fractions: bottom ash (WBA) that is collected under the boiler grate and fly ashes (WFA) collected on a cyclone filter and electrostatic filter. The basic composition of WA depends on the type of biomass, and for the part that is being burned, it depends on ground type or climate [9].

## 2. State of the Art

One of the first waste materials that began to be used in construction was coal fly ash (CFA), produced by the combustion of coal in thermal power plants. Depending on the type, CFA found numerous and diverse applications in cement stabilized mixtures [4,10,11,12,13,14,15,16,17,18,19,20,21,22,23,24]. Unlike stabilization with cement, in which the basic granular material is stabilized instantly, stabilization by materials with pozzolanic properties, such as CFA, is based on the positive effects of delayed pozzolanic reaction.

Characteristic of stabilization mixtures with CFA, in comparison with mixtures with pure cement (CSM), is a gradual increase in strength which also continues for a longer period of time [1,7,11]. Namely, the increase in compressive strength of cement stabilized mixtures (CSM) is intensive during the first 28 days of curing, and it slows down after that. In the case of CFA stabilized mixtures, the development of compressive strength during the first 28 days of curing strength is slightly lower; however, with prolonged curing time (90 days or more), strength may be higher than CSM strength. This can be explained by the delayed pozzolanic activity of this material [1,7,10]. The overall developed heat of hydration in the mixture is, as a rule, reduced with the addition of CFA, which reduces the appearance of thermal cracks from shrinking in the constructed layer [11,12]. A unique phenomenon in stabilized layers is the ability of self-healing—meaning the ability of these layers to activate a mechanism of healing and rebinding of cracks, i.e., damages even after the cracking of a layer (whether the cracks arose due to the characteristics of a semi-rigid layer or whether the damage was caused by external force) [7]. This mechanism of self-healing is the result of the continuing pozzolanic reaction between the activator and CFA in the stabilized mixture. All the mentioned positive effects of CFA have made this industrial by-product the subject of multiannual and numerous studies in mixtures of cement stabilized sand [4,7,10,11,12,13,14,15,16,17,18,19,20,21,22,23,24].

With time, a possible application of other types of ash also started to be investigated in stabilized mixtures, such as ash from municipal waste incinerators [25,26], ash from paper mills, and particularly, bio ash of varying origin and of various fractions [27,28,29,30,31,32,33,34,35,36,37,38,39,40]. In the load-bearing layers of the pavement structure, to improve the load bearing capacity, finer (fly ash (WFA)) and coarser (bottom ash (WBA)) wood ash fractions can be used in two modes of action, depending on their properties. If inert and unable to react on its own, and given its fine granulation, it can be used as filler for mechanical stabilization. On the other hand, WA with a favorable physiochemical composition can be used as a binder if hydraulic or pozzolanic properties are present [41]. According to the research done by Sigvardsen et al. on WA [41,42,43], WA can show pozzolanic activity when high silicone, aluminum, and iron oxide content is present, but in more cases it has been recorded that, unlike the above mentioned CFA, WA are scarce in those pozzolanic minerals and have high calcium content. According to this oxide content, WA are more likely to show hydraulic properties, i.e., to be able to chemically react with water, set and harden, and retain strength and stability under water [43]. Keeping in mind this property and considering the application in roads, other research on this topic is described in more detail below. The stabilization effect of 10% and 20% WFA in mixtures with sand [27] and the unbound pavement of a forest road executed from crushed stone or gravel [28] was investigated by Škels et al. The results showed a multiple increase in load bearing capacity of the mixture expressed by the CBR bearing ratio in both stabilized mixtures, without the appearance of significant swelling. WFA was characterized by the authors as a good independent hydraulic binder for sand stabilization. Vestin et al. [29] investigated the possibility of the application of WFA from burning tree bark for stabilization of a base layer of gravel. Mixtures with 20% and 30% WFA had approximate initial strengths of 4.7, i.e., 4.4 MPa. An increase in compressive strength in the mixture with 20% ash amounted to 2.0 MPa, and in a mixture with 30% ash, it amounted to 5.3 MPa. The same authors reported about the stabilization of a test section with 30% WFA, which resulted in an increase in the load bearing capacity over time. The authors Bohrn and Stampfer [30] reported an increase in the modulus of elasticity E by 20.0 MPa by stabilization of the load-bearing layer of an existing forest road with WBA, in relation to the values obtained by mechanical stabilization of materials. Kaakkurivaara et al. [31] compared the effect of restructuring on test sections with the addition of a new surface layer of gravel (on the reference section) with sections on which, as well as gravel surfacing, the load-bearing layers from WFA or a combination of WFA and gravel were also constructed. An increase in the load bearing capacity was recorded in sections with WFA, but it was less than expected. The greatest improvement of the load bearing capacity was noticed on the sections that had the load-bearing layer made of a mixture of WFA and gravel. The authors attributed poorer results to insufficient compacting during execution, poor technology for mixing of the materials, and different times of storage of ash of particular sections. Sarkkinen et al. [32] ascertained that WA could also be used as a binder for stabilization of granular stone material from dolomite rock waste. The mixtures that consisted of ash from a wood fired power plant, peat and waste from a paper mill, and waste stone material in the proportion of 20:80 had a uniaxial compressive strength of 2.85 MPa after 7 days and 7.3 MPa after 28 days. This mixture, without additional additives, meets the strength properties necessary for installation in load-bearing layers in Finland. More recently, Cherian and Siddiqua [44] researched stabilization of silty sand with the addition of pulp mill (wood) fly ash (PFA). They showed that this type of ash in amounts of 20–30% acts as an effective stand-alone binder for weak subgrade stabilization. It improved strength, resulting in unconfined compressive strength of around 1.6 MPa at 28 days. However, no significant strength gain was recorded with prolonged curing at 60 and 90 days. Cabrera et al. [33] used WBA as a component in cement stabilized load-bearing layers from natural or recycled aggregate. They used a dose of WBA in the contents of 15% and 30%, while the content of cement amounted to 3% and 5%. The research showed that using an amount of WBA up to 15% increases the compressive strength, indirect tensile strength, and the modulus of elasticity of all the mixtures with this WBA in comparison to those that did not contain it, while using a dose of 30% WBA caused a reduction in the modulus of elasticity and indirect tensile strength.

In numerous studies of various types of WA carried out so far, the potential of its application in stabilization mixtures has been confirmed [36,37]. By applying WA, it is possible not only to improve the physical and mechanical properties of the stabilized mixture but also to reduce the proportion of cement in the mixture, which, along with the reduction in WA in landfills, is encouraged by the guidelines of sustainable development [38]. In addition to the numerous advantages of using WA, it should not be forgotten that any application of WA, given that it is a waste material of a variable chemical composition, should be confirmed by conducting complete laboratory tests [39,40].

## 3. Objective of This Study

According to Demirbas [45], biomass, as a renewable energy source, is considered one of the most diverse and valuable resources in the world. According to [46], producing energy from biomass during combustion results in a quantity of ash between 2.7% and 3.5%, on average, of the original weight of wood biomass.

Currently, numerous countries in the world (including Croatia) are faced with ever increasing quantities of bio ash at landfill, which will continue to be “produced” and disposed of, and the reuse/recycling of bio ash is strongly encouraged [9,37]. Unlike other countries, in Croatia, research into the application of WA started to be carried out only in recent years, initiated by large quantities of WA being produced in newly built power plants [47,48,49,50,51,52,53]. WA is produced in an amount of approximately 3.1% of the combusted biomass or about 25,414 tons per year in Croatia. According to the total power of plants which use biomass for energy production, the estimated amount of WA in Croatia is 38,461 tons per year [48]. The passing of the Energy Development Strategy of the Republic of Croatia intensified the construction of power plants on biomass fuels and the first biomass co-generation plant was commissioned in 2011. Almost half of the total number of commissioned power plants are in eastern Croatia, and the largest ones use wood biomass for energy production. The research on the possibilities of recovery of local WA is emphasized by the fact that the Osijek-Baranya and Vukovar-Srijem counties in eastern Croatia are part of the SRCplus project [47]. The European Commission initiated this project in order to produce sufficient quantities of wood chips to meet the national and European energy goals. The project planned the development of a sustainable supply of local power plants/co-generation plants with wood biomass that would come from short rotation, woody crops grown on the agricultural land of poorer quality.

However, even though WA is being progressively produced, its usage in road construction is still the area that requires more research before wider application. While there is some research on the application in asphalts or subgrade improvement, application in hydraulically bound base courses is sparse, considering WA varying properties. Its usage in combination with or as an improvement to local materials of lower quality (sand) for usage as base courses is even less known and therefore the subject of this research. The aim of the research described in this study is (1) to determine, by carrying out the planned tests and analysis of the results, the possibility of using WFA in the load-bearing layers of the pavement structure, while achieving the required and prescribed engineering properties of mixtures. Furthermore, the aim is (2) to enable the rationalization of costs of construction of the pavement structure by using local materials (natural; sand and waste; WFA) and ultimately, in accordance with the concept of sustainable development, (3) to contribute to the reduction in WFA in landfills.

## 4. Experimental Section

**Figure 1:**
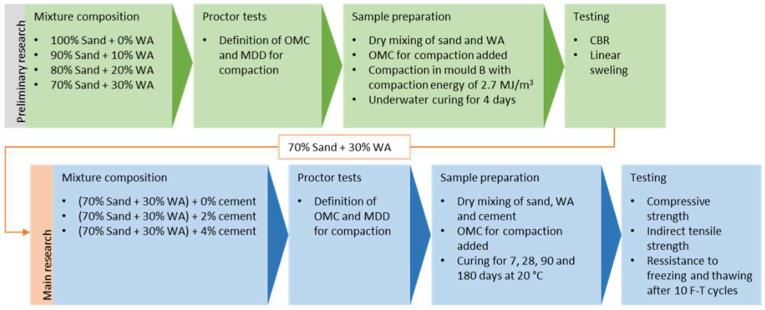
Flow chart of experimental research.

When using new and alternative materials in road construction, all aspects of their application should be determined. Along with the effect on mechanical properties in a given desired application, both the durability and the effect on the environment should be determined. However, determination of such mechanical properties is the first step to check if the application of the material complies with regulation and if further evaluation is justified. In technical regulations, the properties of cement stabilized mixtures are mostly defined by compressive and tensile strength. For cement stabilized materials (CSM), it is usual to test compressive strength after 7 and 28 days of curing of samples, while the period to achieve the required compressive strength may be even longer for materials with pozzolanic properties with a prolonged binding time. In addition to strengths, it is important to know the resistance of stabilized mixtures to freezing, since low environmental temperatures in combination with water can cause additional stress in the cement stabilized layer and also a loss in the load bearing capacity during thawing. In the text that follows, the article describes the tests of the mechanical properties of stabilized mixtures of sand, WFA, and cement: the compressive and indirect tensile strength and the resistance of mixtures to freezing, as is shown in Figure 1.

Laboratory research was carried out on mixtures composed of sand from the Drava River as the basic aggregate, WFA from a co-generation plant for the production of electricity, and heat based on the combustion of wood biomass in eastern Croatia, with cement CEM II/B-M (P-S) 32.5 N and water.

**Figure 2:**
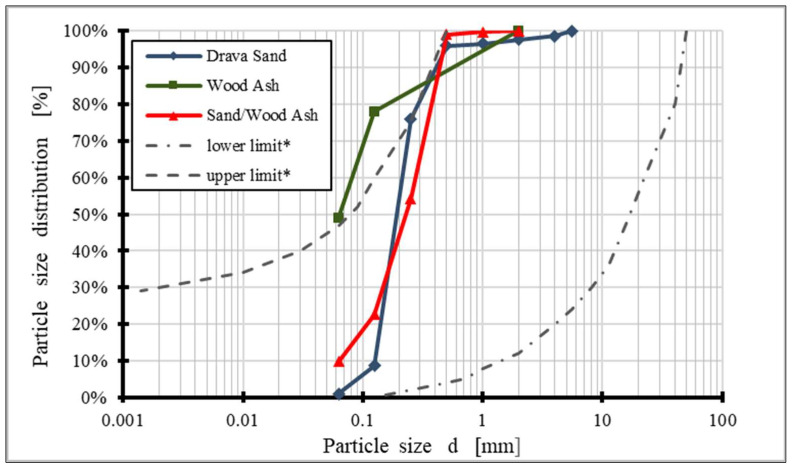
Particle size distribution curves, (* curves of the boundary area are defined according HRN U.E9.024 [55]).

Sand from the Drava River is a material that has proven its application in the construction of embankments and load-bearing layers of pavement structures, although it is a uniformly graded material (SP) that is somewhat harder to compact due to its composition [5]. Determination of the granulometric composition of Drava sand was carried out on samples of 500 g in accordance with the standard HRN EN ISO 17892-4 [54] on a mechanical vibrating table with a set of sieves whose openings ranged from 31.5 to 0.063 mm. According to the sieving results, 96% of the sand particles have a diameter of less than 0.5 mm, and almost 70% of them have a diameter of less than 0.25 mm. The diameter of the largest grain is 1.50 mm, and the proportion of particles smaller than 0.063 mm is under 1% of the mass. Table 1 shows the physical and mineralogical properties of the sand and Figure 2 its particle size distribution. In the granulometric diagram, curves of the boundary area for the application of sandy material in load-bearing layers with stabilized cement have been added, as defined by the Croatian standard for the production of cement-stabilized load-bearing layers, HRN U.E9.024 [55].

**Figure 3:**
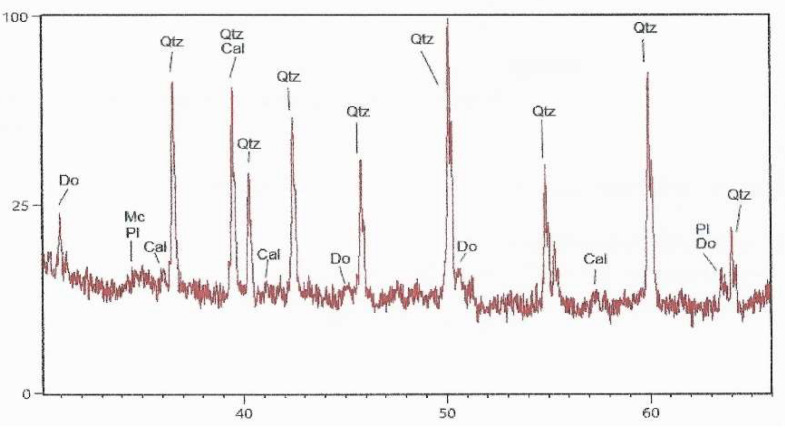
The results of the XRD analysis of Drava sand (Cal—calcite, Ch—chlorite, Do—dolomite, Kfs—potassium feldspar, Mc—mica minerals, Ph—plagioclase, Qtz—quartz) [56].

Mineralogical composition of sand was determined by the qualitative and semi-quantitative X-ray diffraction analysis [56]. For XRD measurement, a PANalytical X’Pert Powder diffractometer was used. X-ray source was CuKα radiation with wavelength of λ = 1.54 Å. The sample was measured in step-scan mode in area between 4 and 66°2θ with step size of 0.02°2θ in sample time of 4 s. The obtained XRD measurements were evaluated by X’Pert HighScore Plus. The mineralogical composition of Drava sand is presented in Figure 3. Results show that quartz is the predominant mineral (71%) and there are high amounts of felspars (24%) in the form of plagioclase ad potassium feldspar. Along with small amounts of calcite and dolomite, there is also a small amount of clay minerals, probably in the form of muscovite and ilite. Even though the main component—quartz—is completely inert and should not contribute to strength or particle packing (uniform sand), components such as feldspar and clay minerals could potentially present a source of aluminosilicates and contribute to strength if activated by source of calcium and water.

**Figure 4:**
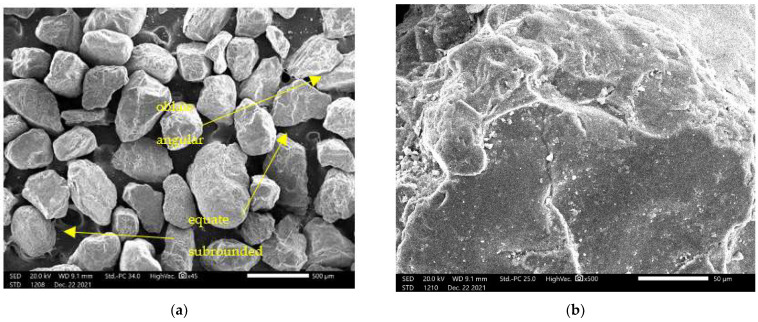
SEM microphotograph of sand: (**a**) magnification SEM_MAG = 45×; (**b**) magnification SEM_MAG = 500×.

As mentioned in [57], both the shape and the texture of sand play an important role in the performance of sand as a pavement material. The shape of the sand particles has a considerable impact on compactness and stability, i.e., the engineering behavior of the sand mixture. Thus, during compacting, irregular sand particles with a rough surface are more favorable than smooth rounded particles and enable a stronger bond to be formed during binding with the cement [58]. Therefore, scanning electron microscopy (JEOL SEM JSM-IT200) was used for images of the shape and size of sand particles (Figure 4). SEM images showed that sand particles vary from oblate to equate with the highest amount of equant, and in terms of the degree of roundness they are angular to subrounded, according to classification in [57], and they have a rough surface.

WFA used in this study is collected on a cyclone filter. The granulometric composition of WFA was determined on sieve openings of 0.063 mm, 0.125 mm, and 2 mm according to the specification Fly Ash for Hydraulically Bound Mixtures (HRN EN 14227-4 [59]) and air jet sieving method was used (HRN EN 933-10 [60]). Granulometric composition of WFA is shown in Figure 2, and its chemical composition is shown in Table 2.

**Figure 5:**
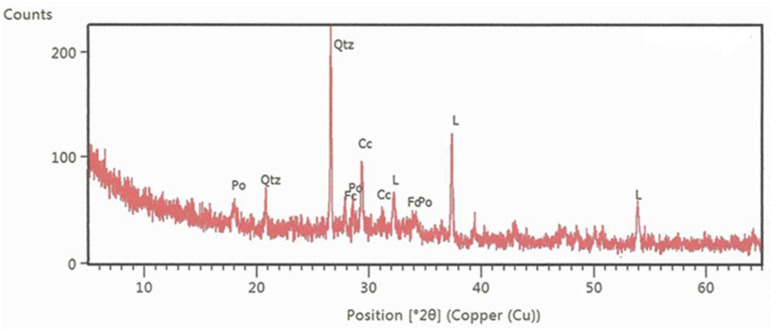
The results of the XRD analysis of WFA [62].

As seen from the composition, the main component is calcium oxide, which indicates that WFA could have hydraulic properties. In turn, content of pozzolanic oxides is quite low and well below the set limit of 70% (EN 450-1 [61]) to be considered source of pozzolan. As a result of testing for the mineral composition of ash samples by the X-ray diffraction method (XRD) [62] (Figure 5), it was determined that the main components of WFA were calcite, quartz, and CaO, and, in smaller quantities, portlandite (CaOH)_2_) and fairchildite (K_2_Ca(CO_3_)_2_).

**Figure 6:**
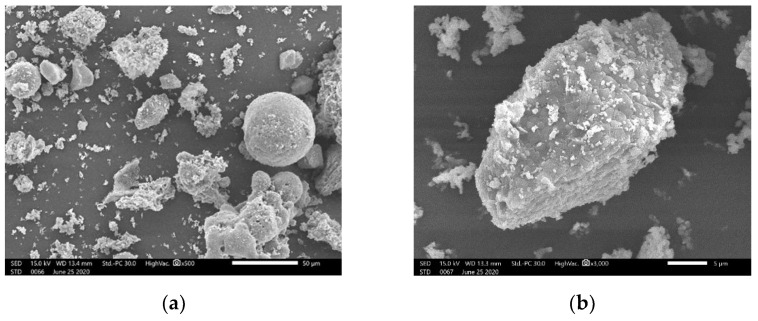
SEM microphotograph of WFA particles: (**a**) magnification SEM_MAG = 500×; (**b**) magnification SEM_MAG = 3000×.

A morphological analysis was carried out using scanning electron microscopy (SEM) (Figure 6.) SEM images show that WFA is composed of particles of different shapes and sizes. Ash particles are irregular in shape and have a rough, porous surface, and, in addition, particle sizes are very uneven. The overall structure of particles does not follow a specific pattern.

Cement CEM II/B-M (P-S) 32,5 N [63] is a type of cement that is most often used for the construction of stabilized layers in eastern Croatia, and its properties are presented in Table 3. For the production of samples of the stabilized mixture, water from the city water supply was used.

### 4.1. Stabilized Mixture Composition

#### 4.1.1. Determination of Optimum WFA Amount in Stabilized Mixtures

The composition of stabilized mixtures is defined, taking into account the fact that WFA can be used in mixtures in two ways: (1) as a binding component, when its addition initiates certain chemical reactions as a result of pozzolanic or hydraulic activity, and (2) as a filler, when it is necessary to improve the physical properties of mixtures by increasing the proportion of fine particles. When initiating chemical reactions in a mixture, the chemical composition of WFA is important, whilst its granulometric composition is also important for improving the physical properties of the mixture.

Since Drava sand is a uniformly graded material (SP), preliminary tests were conducted on mixtures of sand with varying amounts of WFA (0%, 10%, 20%, 30%) as means of mechanical stabilization (Figure 1). The tests included the determination of the optimal moisture content (OMC) and the maximum dry density (MDD), as well as the load bearing capacity of the mixture expressed in accordance with the California Bearing Ratio (CBR). Firstly, sand was dried at 105 °C and both sand and WFA were sieved on a 2 mm sieve opening to remove any larger materials such as shells, rocks, branches, bark, or any unburnt material in ash. No other conditioning was done on those materials. Prior to sample preparation for the test, first the dry sand and WFA were thoroughly mixed, and then the different water amounts were added and mixed until uniform mixture was achieved. Five samples of each mixture were than compacted with energy of modified Proctor test (2.70 MJ/m^3^) in mold A (h = 120 mm, d = 100 mm). After that, samples were weighed and dried to determine the OMC and MDD according to EN 13286-2 [64]. Such determined OMC were then used to prepare samples of all four mixtures for CBR tests. Three samples of each mixture were prepared in a Proctor’s cylindrical mold B with a diameter of 150 mm and a height of 120 mm. After compaction, the samples were submerged in water with a preload of 4.5 kg for 4 days, and the daily reading from the micrometer was recorded so that the increase in linear swelling could be seen. Determination of the CBR ratio on mixtures of sand and WFA was carried out in accordance with the 13286-47 [65] standard, where CBR 1 and CBR 2 are defined by measuring the forces at piston penetration of 2.5 mm and 5 mm and expressed as ratios of standard forces od 13.2 kN and 20 kN, respectively. CBR results are presented in Table 4.

The conducted tests proved that WFA has a direct impact on the increase in the load bearing capacity of the mixture. The CBR ratio increased considerably with the increase in the content of WFA (10%, 20%, 30%) in the mixtures, in relation to pure sand. The highest value of load bearing capacity was recorded for the mixture with 30% WFA and it amounted to 90.70%, which is three times more than bearing ratio of Drava sand (27.44%). This is the result of mechanical stabilization as added finer WFA acted as filler for pore in uniformly graduated sand. However, the samples with highest ash content were also quite cohesive and hardened, which proved that the bearing capacity increase is not a result of better compaction solely and that some form of hydraulic binding occurred. For this reason, in order to carry out further research, a mixture of sand and 30% WFA was chosen as the basic mixture to which cement is added in different proportions.

#### 4.1.2. Designing the Composition of Stabilization Mixtures

The usual proportion of cement required to stabilize Drava sand and achieve the required values of compressive strength in previous tests was 8–12% [5,6]. Considering the WFA content in the mixture and its hydraulic activity potential, and based on preliminary tests, the cement content in the mix was significantly reduced compared to the usual percentage. The aim was to determine the minimum proportion of cement in the stabilized mixture that would ensure that the mixture had the prescribed strength values and adequate resistance to freezing. Mixture composition is visible in Table 5.

A modified Proctor experiment was conducted in accordance with the HRN EN 13286–2 standard [64]. Five samples of each mixture with various moisture contents were prepared to determine OMC and MDD. For the purposes of the research, a Proctor’s cylindrical mold A with a diameter of 100 mm and height of 120 mm was used. Five layers of samples were compacted with the appropriate energy (2.7 MJ/m^3^) in an automatic Proctor device. The resulting values of the OMC and MDD (Table 5) were used to prepare the samples by the same method; they were wrapped in cling film and cured 7, 28, 90, and 180 days in climate chambers at 20 °C. Three samples of each mixture were used to determine compressive strength, indirect tensile strength, and the mixture’s resistance to freezing and thawing.

## 5. Test Methods

### 5.1. Compressive Strength Test

Compressive strength is calculated as average stress of three samples of the same mixture that are exposed to uniaxial pressure at fracture force. The compressive strength of the samples is determined in accordance with the standard HRN EN 13286-41 [66], usually after 7 and 28 days of curing. Each sample is loaded evenly between two slabs, continuously and without jerking with constant increase in force until the samples break. Thus, the maximum load of the sample up to breaking point is recorded and the compressive strength of the sample is calculated according to the formula:

(1) $$f_{c}\;=\frac{F}{\;A}$$

where: f_c_ = compressive strength (MPa); *F* = max compressive force (MN); *A* = sample cross section area (m^2^).

**Figure 7:**
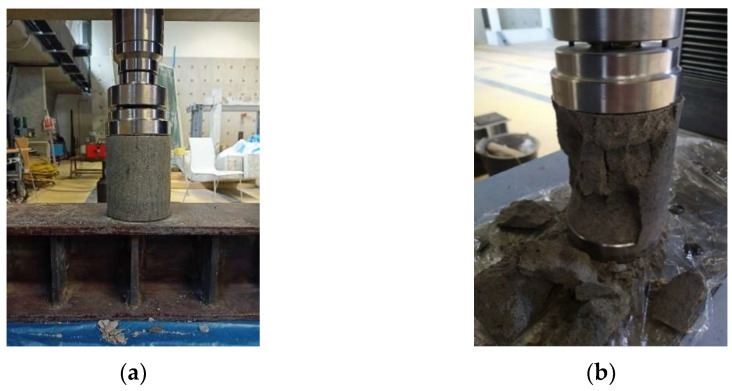
Compressive strength test: (**a**) before loading and (**b**) after loading.

The standard HRN EN 13286-14 [66] also prescribes that the breakage of the sample/appearance of cracks occurs from between 30 and 60 s from starting to apply the load. After breakage, the sample is taken out of the press and types of breakages are studied, which may be satisfactory or unsatisfactory. Testing of compressive strength was carried out by the device Shimadzu Autograph AG-X Series (Figure 7), and the results were processed by the computer program TRAPEZIUM MX.

Testing of compressive strength of the samples was carried out after curing samples at a temperature of 20 °C for 7, 28, 90, and 180 days for mixtures 2 and 3, while for mixture 1 (0% cement), testing of compressive strength was carried out after 7, 28, and 90 days. After curing, the samples were first unwrapped and testing was conducted at room temperature between 21 and 24 °C. Compressive strength was calculated as mean values of three samples for each mixture and curing length. The results of compressive strength testing are presented in Section 6.1.

### 5.2. Tensile Strength Test

Tensile strength was determined by the indirect test method in accordance with HRN EN 13286-42 [67], which is considered the most appropriate for practical testing of cement stabilized materials. Loading is applied on a cylindrical sample by a wood loading bar. Such a load causes a relatively uniform stress perpendicular to the diameter plane at which the pressure is applied. Indirect tensile strength was calculated according to:

(2) $$f_{t}\;=\frac{2F}{\pi hd}$$

where: f_t_ = indirect tensile strength (MPa); *F* = max compressive force (MN); *h* = length of the sample (m); *d* = diameter of the sample (m).

**Figure 8:**
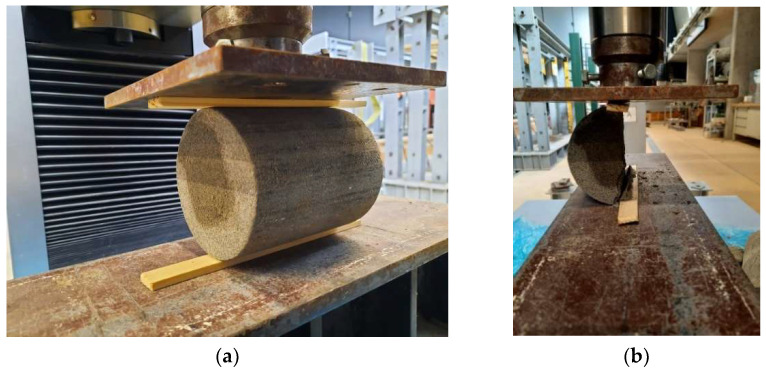
Indirect tensile strength tests: (**a**) before loading and (**b**) after loading.

Testing of indirect tensile strength of the samples was carried out after curing samples at the temperature of 20 °C for 7, 28, 90, and 180 days, except for the samples of mixture 1, on which testing was carried out after 7, 28, and 90 days. As in compressive strength, samples were first unwrapped and then tested at room temperature, while each indirect tensile strength result shown is calculated as mean of three tested samples. The equipment used in this testing is the same as that used for testing of compressive strength with the exception of the connection through which the load is applied (Figure 8). The results of indirect tensile strength testing are presented in Section 6.3. 

### 5.3. Testing of Resistance to Freezing and Thawing

**Figure 9:**
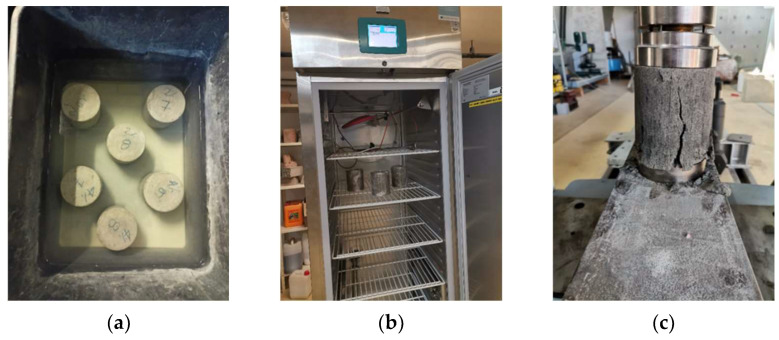
Testing of resistance to freezing and thawing: (**a**) two-day care of samples under water; (**b**) climate chamber; (**c**) compressive strength test after freezing/thawing cycles.

The resistance of samples to freezing was tested in accordance with the European technical specification HRS CEN/TS 13286-54 [68] according to which the resistance of mixtures to freezing and thawing is defined by measuring the compressive strength of the samples that have been subjected to freezing and thawing cycles and the control samples that were cured for the same period according to the prescribed curing regime. Two sets of samples, with the same dimensions and shape as for compressive strength testing, were prepared, wrapped in plastic adhesive foil, and cured for 28 days at a temperature of (20 ± 2) °C. The technical specification HRS CEN/TS 13286-54 [68] prescribes that the samples should be in this regime of curing for 28 days during which strengths develop, and then followed by a two day (secondary) curing of the samples under water, in a water saturated climate chamber or curing the same as in the first 28 days. In this research, the form of curing least favorable for the samples was used, i.e., two-day curing under water (Figure 9a). This method of curing enables direct contact of water with the sample and the action of water during freezing of the sample. After the two-day curing under water, Set A of each mixture was subjected to freezing and thawing cycles in the climate chamber (Figure 9b) while Set B—the control set—was left at the secondary curing during this period. Each cycle of freezing lasted 24 h and consisted of a period during which the temperature dropped from +20 °C to −18 °C, which was then maintained for a further 6 h, and then a period of thawing. After 10 freeze/thaw cycles, the samples were returned to the original cure regimen with a control set of samples for 24 h, and then both sets of samples were subjected to compressive strength testing (Figure 9c). Considering that significant quantities of salt are spread on road pavements in eastern Croatia during the winter period, an additional freeze/thaw test was carried out with the presence of salt, in accordance with EN 1367-1 [69] (Set C). The water, in the second stage of curing, was replaced with a solution of 1% sodium chloride (NaCl).

Retained compressive strength after freezing was calculated according to the formula:

(3) $$\mathrm{RFT}=\frac{M_{A}}{M_{B}}*100$$

where: RFT = retained strength factor, after freeze/thaw testing, *M_A_* = the mean value of strength for Set A (MPa), *M_B_* = the mean value of strength for Set B—control (MPa).

## 6. Results Analysis and Discussion

### 6.1. Analysis of Compressive Strength Results

**Figure 10:**
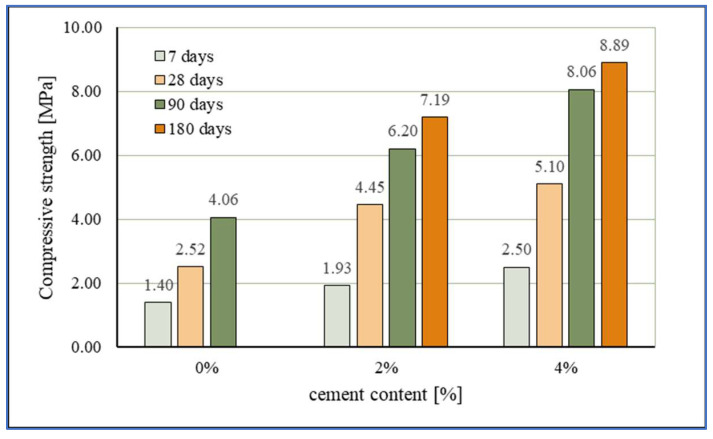
Effect of the cement content on the compressive strength.

**Figure 11:**
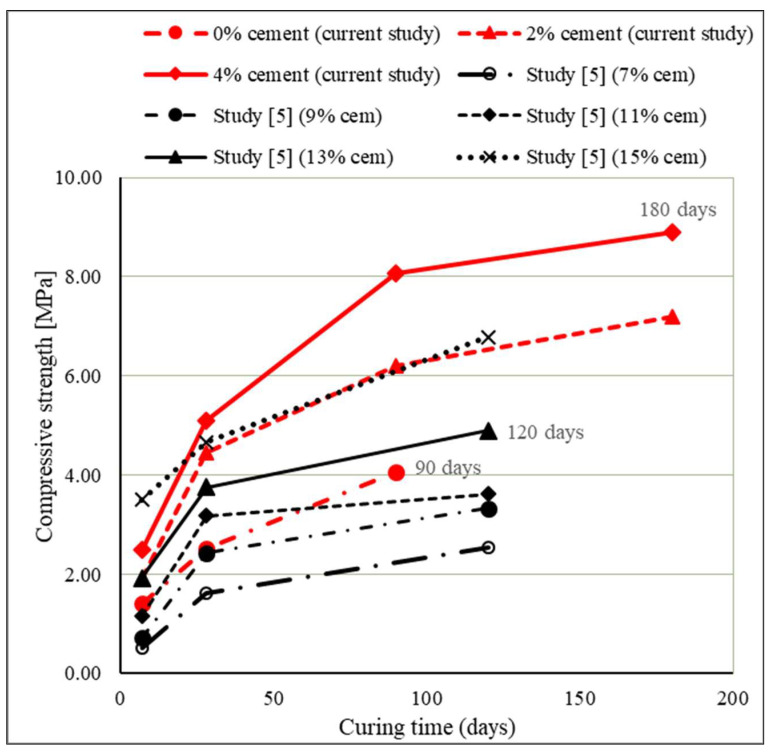
Effect of curing time on the compressive strength.

**Figure 12:**
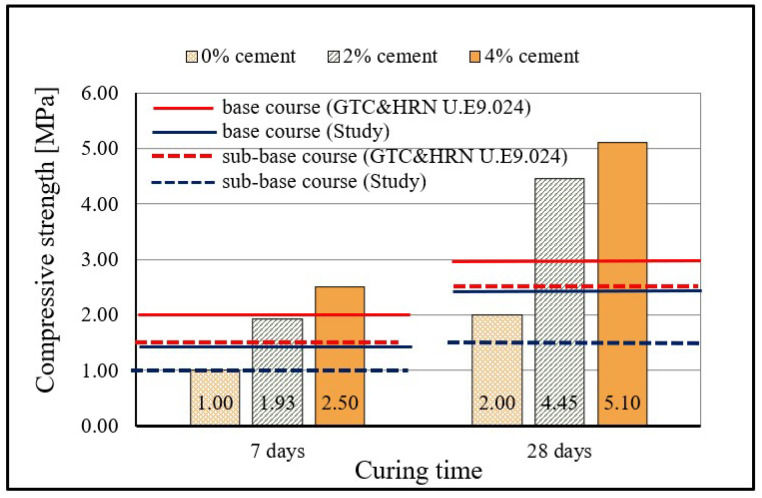
Comparison of compressive strengths of mixtures with the prescribed criteria of the Croatian technical regulation [5,55,77].

The compressive strength results of stabilized mixtures are presented in Figure 10 and Figure 11 and the development of compressive strength over time compared with the results from the study [5] is presented in Figure 12.

The stabilized mixture with 0% cement had the following compressive strength values: 1.4 MPa after 7 days, 2.52 MPa after 28 days, and 4.06 MPa after 90 days. By adding a very small quantity of the cement, the reaction is even more intensive and results in higher strength values. Thus, the mixture with 2% cement on average had 55.7% higher compressive strength than the mixture with 0% cement; the greatest increase in strength recorded between mixtures was after curing for 28 days. The highest values of compressive strength were, as expected, obtained by mixtures with 4% cement: 2.5 MPa after 7 days, 5.10 MPa after 28 days, 8.06 MPa after 90 days, and a value of compressive strength of 8.89 MPa after 180 days. The average increase in compressive strength of mixtures with 4% cement compared to mixtures with 0% cement is as much as 75.7%, and in this case the largest increase in compressive strength (102.4%) was recorded after curing for 28 days.

Although the bearing capacity (CBR) improvement can be attributed to the change in particle size distribution of sand by the addition of WFA, the compressive strength development in mixtures without cement points to some form of hydraulic hardening as well. According to some authors [43], this is sometimes due to WFA acting as a pozzolanic binder but more commonly it is due to WFA acting as a hydraulic binder. The WFA used here contains only a small amount of pozzolanic oxide but it has large amounts of calcium oxide. Even though total oxide content does not necessarily predict the actual performance and WFA has shown only low self-cementing properties (per ASTM D5239 [70]), recorded compressive strength on mixtures without cement is significant. As WFA could not on its own develop this compressive strength, results obtained on mixtures with no cement (0%) must be the product of the interaction of WFA and sand, i.e., their chemical and mineralogical compounds. This WFA that has high CaO content, in the mixtures with presence of water, creates a highly alkaline environment (pH of 12.7 [71]). As in the lime application, Ca+ and OH- ions could also initiate chemical reactions if aluminosilicates were available and this would result in the development of the compressive strength. Given that the major constituent of sand is quartz (71%), which is generally not reactive, an explanation could come from the joint effect of the CaO reaction with the small number of clay minerals in sand, the low SiO_2_ content of WFA, and also the feldspar minerals in sand (25% of sand content). The pozzolanic reaction of lime with clay minerals that results in hydration products, such as in cement hydration (CSH and CAH), is well known [72]. However, at 1% content of clay minerals in sand, it can hardly be responsible for all the gained strength. However, feldspar is also an aluminosilicate with a chemical composition that includes Ca, Si, and Al cations and, therefore, pozzolanic properties could be expected [73]. Because around 80% of feldspar is made from SiO_2_ and Al_2_O_3_, the authors in [74] believe that in their research it reacted directly with Ca(OH)_2_ available from cement to form a hydration product such as CSH. According to [75], lime added to properly treated feldspar can create an agent that can act as an activator for the development of pozzolanic reactions in known silica sources such as slag and fly ash of silica fume. This should result in material with cementitious composition that can be used in filings, paving, or stabilizations.

In this research, sand containing feldspar minerals was mixed with WFA that had high CaO content, and this resulted in hydraulic activity-mixture hardening and strength development. It is reasonable to assume that the gained compressive strength of the mixture without cement can be attributed to the alkaline environment created by the WFA addition that then led to a hydraulic reaction with the available silica and the development of cementitious hydrates. However, even though measured mechanical properties point to this, further research into the developed mineralogic phases, which was not in the scope of this study, is necessary to fully understand these effects.

The addition of 2% and 4% of cement to mixtures further increases their strength. By increasing the cement content in the mixtures, the compressive strength value increases—mainly due to the hydraulic reactions between cement components (lime and aluminosilicates), which leads to the production of hydrated calcium silicate (CSH) and hydrated calcium aluminate (CAH).

The binding activity continued over time, which is evident by the increase in mechanical properties (compressive and tensile strengths) of the samples over time. However, since cements are formulated such that most of the compressive strength gain is achieved in the first 28 days, the significant long term strength gain recorded here (90 and 180 days) could be due to slower pozzolanic reaction. Pozzolanic reactions are well known to occur over longer time periods, such as months or years, in normal curing conditions. The mechanism of strength gain involves using available and unused Ca(OH)_2_ in moist conditions (presence of water) to activate the source of pozzolans and to create further cementitious materials (CSH and CAH) through time. This mechanism is particularly noticeable in the diagram in Figure 11, where the contribution of WFA achieves higher values and the development of compressive strength of the mixture over time is clearly seen.

Increasing the curing time of the specimens had a positive effect on the increase in compressive strengths of the mixtures. From the results, it can be seen that the increase in compressive strength in mixtures with 2% and 4% cement, although greatest during the first 28 days of curing (130.57% for 2% cement and 104% for 4% cement), is significant and continuous for longer periods of curing. Namely, in addition to the relatively rapid increase in compressive strengths during the first 28 days of curing due to hydraulic reactions, long-term pozzolanic reactions also develop. A particularly significant increase in compressive strength over time was achieved by mixtures with 0% cement with WFA only, taking into account the properties of sand and WFA and necessary prolonged curing for the pozzolanic reaction. Mixtures with WFA achieved 80% higher compressive strength after 28 days and 190% higher strength after 90 days (compared to 7-days compressive strength) and thus confirmed the ability of WFA to act as a binder in the sand mixture. The test results show that, by replacement of part of the sand with WFA, in the quantity of 30%, much higher values of compressive strengths can be achieved than in mixtures of sand stabilized only with cement. This is particularly evident in Figure 11 where the obtained results are compared with the results from the study [5], in which compressive strengths of stabilized mixtures composed of Drava sand and cement were analyzed. Cement that was then used in testing had the same properties and class as the cement that was used in the research described in this article. The determination of compressive strength was conducted after 7, 28, and 120 days. The measured compressive strengths were 0.5 MPa and 1.62 MPa for the mixture with 7% cement; 0.71 MPa and 2.43 MPa for the mixture with 9% cement; 1.15 MPa and 3.18 MPa for the mixture with 11% cement; 1.925 MPa and 3.75 MPa for the mixture with 13% cement; and finally, 3.5 MPA and 4.67 MPa for the mixture with 15% cement, measured at 7 and 28-days curing, respectively.

Comparing the results obtained in this study with the results of the study [5], a significant difference in the values of compressive strengths can be seen, i.e., the significant contribution of WFA in the mixture to the increase in compressive strengths. Thus, a mixture of sand and WFA with 2% cement and mixture of pure sand and 13% cement had the same compressive strengths at 7-days curing, while higher compressive strength was measured at 28-days curing on the mixture with WFA and 2% cement (4.45 MPa vs. 3.75 MPa). The mixture of sand and WFA with 4% cement achieved a 7-day compressive strength (2.50 MPa) greater than the mixture of sand stabilized with (as much as) 13% cement (1.925 MPa). The most interesting result is certainly that of the mixture with 0% cement which (with only WFA) achieved a higher compressive strength already after 7 days (1.40 MPa) compared to mixtures of sand stabilized with 7%, 9%, and 11% cement. In addition, for this mixture without cement, an intensive increase in compressive strength is noticed even after 28 days, which was less prominent for mixtures of sand and lower cement content from the study [5]. The stabilized mixture with 2% cement achieved higher values of compressive strengths from all the mixtures of sand and cement, except the mixture with 15% cement. The stabilized mixture with 4% cement had a lower value of compressive strength (2.50 MPa) after 7 days than the mixture of sand and 15% cement (3.50 MPa); however, over time, already after 28 days, the mixture with 4% cement achieved a compressive strength of 5.10 MPa and the strength continued to increase with the duration of curing. Clearly evident in the diagram is the exceptional contribution of WFA in the mixture of sand to the results of compressive strength, as well as the continuous development of compressive strength over time. Additionally, the results confirm the conclusions of previous research [11,16,18,33] that the compressive strength in mixtures with fly ash (with a notable content of CaO) and cement is considerably higher than the compressive strength of mixtures stabilized with cement only.

The Geotechnical Classification of NRC materials, suggested by the author in [76] (NRC: New construction based on recycled materials) classifies mixtures into five groups (A–E) according to the minimum compressive strength. The materials, i.e., mixtures that achieve a minimum compressive strength of 4.5 MPa belong to Group A (very strong), while Group E is for materials in which the development of strength does not occur (no strength development). According to the proposed classification, stabilization mixtures with 0% and 2% cement belong to Group B: strong (min. 1.5 MPa) while the mixture with 4% cement belongs to Group A: very strong (min. 4.5 MPa); therefore, they could be used as bearing layers.

### 6.2. The Comparison of Values of Compressive Strengths of Stabilized Mixtures with the Criteria Prescribed by the Applicable Croatian Technical Regulations

In order to determine a possible application of stabilized mixtures in the construction of stabilized load-bearing layers, the results of compressive strength after 7 and 28 days must meet the criteria prescribed by the standard or technical conditions. There are still no precisely defined requirements regarding quality and the load bearing capacity of the layers stabilized with fly ash (or any other pozzolan) in the Republic of Croatia, and the comparison of the results obtained will be carried out on the basis of the criteria applicable for mixtures stabilized with cement.

As already stated in the introductory part of the study, for the needs of construction of pavement structures with sand in load-bearing and stabilized layers, the *Study of the possibilities of the application of sand in the construction of roads of Slavonia and Baranya region* [5] was prepared. The study [5] prescribed somewhat lower criteria of compressive strength in relation to the criteria defined by the HRN U.E9.024 standard [55]. The standard does not differentiate between the type of aggregate in the load-bearing layer (sand, gravel, or crushed stone) and the values of compressive strengths are defined in relation to the road category (i.e., the expected traffic load) and the position of the layer in the pavement structure. Therefore, the conditions for the application of mixtures of sand are stricter. Non-standard binders such as fly ash or slag are mentioned only in the General Technical Requirements for Road Work (GTR) [77]. The GTR stipulates that when using binders other than cement (fly ash, slag), the compressive strength limits of the mixture remain the same as for cement but with a prolonged duration of curing that needs to be determined on the basis of laboratory tests. The defined minimum compressive strength criteria and the results of the comparison are shown in Figure 12.

From the diagram in Figure 12, it is evident that the mixture with 4% cement meets all the criteria for application, regardless of the position of the layer in the pavement structure. These results are particularly good when we take into account that the criteria for application are defined for mixtures of crushed stone or gravel. The mixture with 2% cement meets all the criteria of application according to the study and it meets the (stricter) criterion for application in lower base layers, according to the standard. Although, after 28 days, the compressive strength of this mixture is more than sufficient for application in upper base layers, the value of the strength of the mixture with 2% cement is somewhat lower than required after 7 days. The mixture with 0% cement, strictly speaking, does not meet the criteria for application in upper base layers and may be used in lower base layers of the pavement structure. However, the achieved results of compressive strength are good, bearing in mind the type of binder in the mixture. Namely, in the case of fly ash, a longer time of curing for the development of compressive strength should be defined, whilst here the results for curing of 7 and 28 days are analyzed, according to the criterion for mixtures stabilized with cement. In the results for curing for 28 days, an improvement of 80% in compressive strength of the mixture with 0% cement is already evident.

### 6.3. Analysis of Indirect Tensile Strength Results

**Figure 13:**
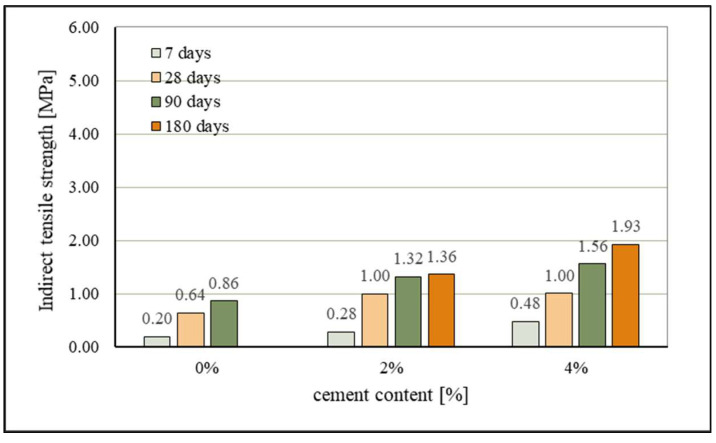
Effect of cement content and curing on indirect tensile strength.

The results of the indirect tensile strength tests are presented in Figure 13. The trend of the development of indirect tensile strength for all stabilization mixtures and all durations of curing is very similar to that for compressive strengths and it is attributed to the same binding effects. The stabilized mixture with 0% cement gave the following values of indirect tensile strength: 0.2 MPa after 7 days, 0.64 MPa after 28 days, and 0.86 MPa after 90 days, which confirms the activity of these ashes as binders in the mixture of sand of this mineral composition. By adding a very small quantity of cement, the reaction was more intense, and the strength results were considerably higher. Thus, the mixture with 2% cement achieved on average (for curing durations of 7–90 days) 49.9% higher tensile strengths than the mixture with 0% cement, with the greatest increase in tensile strength recorded during the curing of 28 days (56.25%). The greatest values of tensile strength were, as expected, achieved by the mixture with 4% cement: 0.48 MPa after 7 days, 1.0 MPa after 28 days, 1.56 MPa after 90 days and, after 180 days, the value of tensile strength was 1.93 MPa. The average increase in indirect tensile strength of mixtures with 4% cement in relation to the mixtures with 0% cement, for curing of 7 to 90 days, amounts to 75.55%, almost the same as in the case of the compressive strengths of the mixture.

With prolonged curing, a considerable increase in indirect tensile strength can also be seen. Thus, for mixtures with 0% cement, the increase after 90 days, in comparison with an initial strength after 7 days, amounts to 330%. Mixtures with 2% cement even achieved an increase in strength of 371.4% after curing of 90 days, whilst the average increase in indirect tensile strength for all durations of curing (7–180 days) was 245.0%. Mixtures with 4% cement achieved a 302% increase in strength after 180 days in comparison to 7 days, while, after 90 days, they had a strength of over 225% in comparison to the 7-day strength. All the obtained results of indirect tensile strengths, as was the case with compressive strengths, are the result of the hydraulic and pozzolanic activity between constituents of the mixture, further improved by the addition of cement, and also somewhat due to the favorable shape and roughness of the surface of the grains of sand [78].

### 6.4. Correlations between Compressive and Indirect Tensile Strength

Regression analysis of the test results enabled the establishment of connections (correlations) between compressive and indirect tensile strengths of stabilization mixtures and the determination of the shape and strength of these connections. The regression analysis conducted was between compressive and indirect tensile strength of the samples of the same mixture and same curing length, for example, between f_c_ and f_t_ of mixture 1 at 28 days of curing. This type of analysis is useful as it enables the prediction of indirect tensile strength of mixtures based on their compressive strength with a high degree of confidence and also shows possible variability of different design mixtures. For each mixture group and all mixtures combined, a linear correlation model in the form of

f_t_ = a * f_c_ + b (4)

was analyzed and coefficient of determination, R^2^, was calculated.

**Figure 14:**
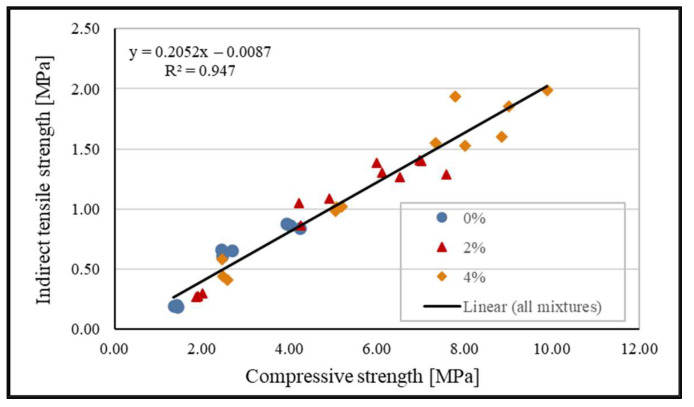
Diagram of the correlation of strengths of stabilized mixtures.

The results of the regression analysis are shown in Table 6 and in the diagram in Figure 14.

The linear correlation between the indirect tensile strength and compressive strength of stabilizing mixtures is very strong as the coefficients of determination R^2^ for all models were between 0.90 and 0.947. The indirect tensile strength of stabilization mixtures with cement amounts to 20% of the value of compressive strength for both contents of cement, and the mixture without cement to 24% of compressive strength. This difference in values of mixtures with and without cement can be explained by the difference in the composition but also by the fact that for the mixture with 0% cement, strengths for 180 days of curing were not determined and therefore not included in the analysis. The average correlation of indirect tensile strength in relation to compressive strength for all the tested mixtures and all the durations of curing amounts to 20%. This mutual strength relationship is higher and differs from the strength results of stabilized mixtures of similar composition [1,7] which range from 10% to 17%. The mentioned differences in strength ratios can be explained by differences in the type of fly ash used as well as differences in the composition of mixtures. This study points out that WA should be further researched as a conclusion on the conventional hydraulically bound mixtures or those with CFA cannot fully be applied on WA.

### 6.5. The Results of Resistance to Freezing and Thawing

**Figure 15:**
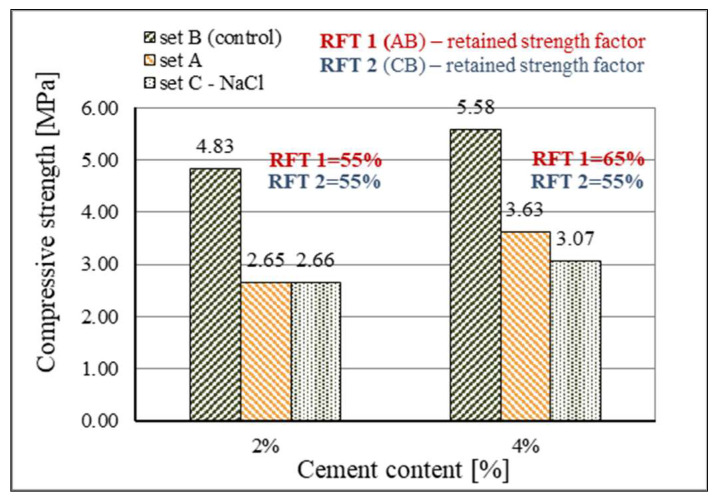
Results of resistance to freezing and thawing of stabilized mixtures.

The results of testing the resistance of mixtures with 2% and 4% cement to freezing are presented in Figure 15. Due to the lower value of initial strengths, testing of resistance to freezing of the mixture with 0% cement was not carried out. The control mixture with 2% cement achieved a compressive strength of 4.83 MPa, while the control mixture with 4% cement achieved a compressive strength value of 5.58 MPa. It can be seen that the increase in content of cement in the control mixtures results in a 13% higher value of compressive strength and strengths here are following those measured at 28 days of curing for compressive strengths tests (4.45 MPa and 5.1 MPa, respectively). In mixtures that were subjected to freezing, the difference in the achieved strengths for different contents of cement is greater.

The mixtures with 2% cement achieved a compressive strength of 2.65 MPa after freezing, which represents a considerable reduction in comparison with the control mixture for which the compressive strength amounted to 4.83 MPa. The retained compressive strength of the mixture after freezing (RFT 1) amounts to 55% of the compressive strength of the mixture before the freezing process. The compressive strength after freezing of the mixture with 4% cement amounted to 3.63 MPa, while compressive strength of the control mixture with 4% cement was 5.58 MPa. For stabilization mixtures with 4% cement, the retained compressive strength after freezing amounts to 65%.

The results of additional testing of resistance to freezing with the presence of salts (RFT 2) showed that the value of retained strength after freezing for both mixtures is identical, regardless of the content of cement in them, and it amounts to RFT = 55%.

The standard [68] does not define the criteria for the satisfactory resistance of a mixture to freezing, but the American standard ASTM C593 [79] precisely defines the resistance of the mixture with fly ash to freezing, prescribing a minimum compressive strength of mixtures in the quantity of 2.8 MPa. Furthermore, Lahtinen [76] states that for the application of stabilization mixtures with fly ash in the base layers of roads with low traffic (low volume roads), the greatest reduction in compressive strength after freezing can be 40%. Besides the prescribed reduction in strength, the same author states that the samples must remain firm and undamaged after freezing.

It should be pointed out that testing of the freezing/thawing of samples simulates conditions that are much more complex than the actual conditions “in situ” and that rarely occur in the field. Stabilized layers are usually in the middle of the pavement structure and are protected by an asphalt or concrete pavement from above, and there are unbound base layers constructed under them, which prevent capillary penetration of water or the rising of the underground water level. In addition, the reduced strength of the mixture after curing in water with a content of salt can be seen in a similar way. Although a smaller quantity of NaCl can appear through cracks in the pavement in stabilized base layers during the winter period, it is not expected that such a quantity of salt will jeopardize the load bearing capacity and durability of the layer.

On the basis of the above, it can be concluded that the mixture with 4% cement has an adequate resistance to freezing, and that it can be used for the construction of base layers of the pavement structure in a region with a continental climate, such as the climate of eastern Croatia. The mixtures with 2% cement (although very similar to the mixture with 4% cement according to the results obtained) did not meet the criteria for satisfactory resistance to freezing.

## 7. Conclusions

In this study, tests on mixtures of Drava sand, WFA, and cement were carried out, which included determining the CBR bearing ratio, the compressive and indirect tensile strength of mixtures, and their resistance to freezing/thawing. The test results obtained show that local materials of lower quality, WA and sand, can be used in hydraulically bound mixtures and they satisfy the conditions given for road base courses of higher quality materials. The following can be concluded:

(1) WFA with a considerable content of CaO (46.91 mass %) has a stabilization effect on the sand mixture and improves the load bearing capacity of the mixture expressed by the CBR index. The CBR index increases considerably with an increase in the content of ash in the mixture (10%, 20%, 30%) both due to the filler effect and through hydraulic binding between minerals in sand and WFA. The greatest value of CBR (90.70%) was achieved by mixtures of sand and 30% WFA, which is three times more than the bearing ratio of pure sand (CBR = 27.44%).
(2) WFA in the mixture of sand improves compressive strength, so that the mixtures with 0% cement achieved compressive strength values of 1.4 MPa (7 days) to 4.06 MPa (90 days). These results indicate the development of hydraulic and pozzolanic reactions between sand and WFA, but further research is necessary to clearly define the reactions and minerals that occur. By adding a small quantity of the cement (2% and 4%), the hydraulic reaction is even more intensive and results in higher values of compressive strength. According to the proposed classification based on a minimum compressive strength for new construction based on recycled materials [76], stabilizing mixtures with 0% and 2% cement belong to Group B: strong (min 1.5 MPa) whilst the mixture with 4% cement belongs to Group A: very strong (min 4.5 MPa).
(3) The comparison of the values of compressive strengths after 7 and 28 days with the criteria of the Croatian technical regulations (prescribed for mixtures stabilized exclusively with cement) showed that the mixtures with 4% cement meet the conditions for application in the upper and bottom base layers of the pavement structure. The mixtures with 2% cement can be used for the construction of the subbase layers, as well as the mixtures without cement.
(4) The trend of the indirect tensile strength development for all stabilized mixtures and durations of curing is very similar to that of compressive strengths. The stabilized mixture with 0% cement achieved the values of indirect tensile strength from 0.2 MPa (7 days) to 0.86 MPa (90 days). The average increase in indirect tensile strength of the mixtures with 4% cement in comparison with the mixtures with 0% cement, for curing of 7 to 90 days, amounts to 75.55%, almost the same value as in the relation of compressive strengths of the mixture.
(5) For compressive and indirect tensile strengths of mixtures, the model of the correlation f_t_ = a * f_c_ + b was analyzed. The results obtained show that the linear correlation between indirect tensile strength and compressive strength of stabilization mixtures is very strong, and coefficients of determination are very high (R^2^ = 0.90–0.947). The average correlation of indirect tensile strength in relation to compressive strength for all the tested mixtures and all the durations of curing amounts to 20%.
(6) The mixture with 4% cement has a satisfactory resistance to freezing, and it can be used for the construction of load-bearing layers of pavement structures in a region with a continental climate, such as the area of eastern Croatia.
(7) In addition to a significant contribution to achieving the required mechanical properties of mixtures, it is necessary to emphasize that the environmental and economic benefits of WFA in load-bearing layers of a pavement structure are reflected in:
  (I) The rationalization of costs of stabilized load-bearing layers through savings in cement and sand amount. WFA is a cost-free material, and with the replacement of 30% of sand with WFA and the decrease in cement content from the standard amount of 8% to 4%, the construction costs of pavement structure are reduced.
  (II) A reduction in the quantity of this waste material at landfills. The daily quantities of WFA that are produced are significant and they are mostly deposited in landfills. Depositing in landfills is demanding and expensive and takes up valuable space, so reuse/recycling is strongly encouraged.
  (III) Protection of natural aggregates. Clear requirements for conservation and protection of non-renewable sources of natural aggregates is stated in the sustainable development guidelines and the usage of WFA means a lower requirement for the exploitation of natural aggregates.

In highlighting the advantages of WFA usage, one should not neglect the fact that WFA is a waste material with variable chemical and mineralogical compositions, and as such, it could have environmental impacts. However, prior to their wider usage in the road construction industry, the durability and environmental effect of such materials should be further investigated.

## Figures and Tables

**Table 1:** Physical and mineralogical [56] properties of sand from the Drava River.

Physical Properties	UCSC	Density (Mg/m^3^)	Color	D_10_ (mm)	D_30_ (mm)	D_60_ (mm)	C_u_	C_c_
	SP	2.68	Grayish-Brown	0.13	0.16	0.22	1.68	0.895
Mineralogical properties	type of mineral	quartz	calcite	dolomite	feldspars	clay minerals		
	mass %	71	2	2	25	1.2		

Note: C_u_ = coefficient of uniformity; C_c_ = coefficient of curvature; D_X_ is the diameter of material particle below which X percent of materials are finer than this D_X_ size.

**Table 2:** Chemical composition of WFA.

Components	MgO	Al_2_O_3_	SiO_2_	P_2_O_5_	SO_3_	K_2_O	CaO
mass %	3.06	0.44	4.05	2.90	1.59	2.82	46.9

**Table 3:** Properties of cement CEM II/B-M (P-S) 32,5 N according to HRN EN 197-1:2012 [63].

Initial Time Setting (min)	Stability of Volume According to Le Chatelier (mm)	Unconfined Compressive Strength (2, 28 Days) (MPa)	SO_3_ (%)	Cl (%)
200	0.4	16.0; 42.0	3.20	0.009

**Table 4:** Results of the CBR ratio and linear swelling of sand/WFA mixtures.

Test		Amount of WFA in Mixtures			
		0%	10%	20%	30%
CBR 1	%	27.44	48.99	52.69	82.09
CBR 2	%	18.96	59.39	56.82	90.70
Linear swelling	%	0.02	0.08	0.22	0.64

**Table 5:** Proctor elements OMC and MDD for sample preparation.

Mix No.	Mixture Composition	OMC [%]	MDD [g/cm^3^]
1	(70% sand + 30% WA) + 0% cem	13.50	1.72
2	(70% sand + 30% WA) + 2% cem	12.69	1.73
3	(70% sand + 30% WA) + 4% cem	11.61	1.70
Drava Sand		14.20	1.64

**Table 6:** The results of the regression analysis.

Mix No.	Days of Curing	Model	Coefficient of Determination R^2^
1	7, 28, 90	a = 0.2428 b = 0.0777	0.9078
2	7, 28, 90, 180	a = 0.2089 b = 0.0411	0.9227
3	7, 28, 90, 180	a = 0.2075 b = 0.0323	0.9435
All mixtures		a = 0.2052 b = 0.0087	0.9470
